# Efficacy of adjuvant immune checkpoint inhibitors pembrolizumab or nivolumab in melanoma patients ≥ 75 years: results of a real-world cohort including 456 patients

**DOI:** 10.1007/s00262-024-03750-1

**Published:** 2024-07-05

**Authors:** A. Gawaz, I. Wolff, L. Nanz, L. Flatz, A. Forschner

**Affiliations:** Universitätshautklinik Tübingen, Liebermeisterstr. 25, 72076 Tübingen, Germany

**Keywords:** Immune checkpoint inhibitor, Adjuvant therapy, Melanoma, ≥ 75 years, Elderly patients

## Abstract

**Background:**

Immune checkpoint inhibitors (ICI) applied in patients with melanoma in an adjuvant setting have proven safety and efficacy in several studies, but data on elderly patients aged 75 years or more is scarce. Aim of this study was to investigate efficacy and safety of adjuvant ICI in patients aged ≥ 75 years compared to patients < 75 years in a real-world setting.

**Methods:**

We retrospectively analyzed clinical data, including occurrence of immune-related adverse events (irAE) and outcome of 456 patients that had been treated with adjuvant ICI between January 1st, 2018 and December 20th, 2022. We then compared patients aged ≥ 75 years (*n* = 117) to patients < 75 years (*n* = 339) in terms of safety and disease-free survival (DFS).

**Results and conclusion:**

ICI were well tolerated in both groups, with no significant difference observed in the overall occurrence of irAE. However, within the elderly subgroup, there was a significantly higher proportion of skin or nephrological toxicity and colitis/diarrhea compared to the other group. In terms of efficacy, a significantly shorter DFS in patients aged ≥ 75 years was observed. Adjuvant ICI in patients ≥ 75 years was less effective and furthermore associated with an increased risk for skin, renal or bowel toxicity. Therefore, in elderly patients, adjuvant ICI should be used with precaution.

**Electronic supplementary material:**

The online version of this article (10.1007/s00262-024-03750-1) contains supplementary material, which is available to authorized users.

## Introduction

Immune checkpoint inhibitors (ICI) have proven to be an effective treatment for metastasized malignant melanoma [1]. Targeting the transmembrane protein Programmed cell death 1 (PD1) on cytotoxic T cells, PD1 inhibitors induce an antitumoral immune response by promoting T cell proliferation and infiltration into the tumor [1, 2]. Since 2019, the PD1 inhibitors pembrolizumab and nivolumab have also been approved in the adjuvant setting of patients with stage III and stage III/IV melanoma. For both ICI, a significant prolonged DFS has been shown in the adjuvant setting [3, 4]. However, treatment with ICI is often accompanied by autoimmunity, which can affect any organ and may result in a variety of immune-related adverse events (irAE) [5].

It is known that tumor thickness and ulceration rate of melanoma increases with age [6]. Therefore, an increasing number of elderly patients with melanoma are offered adjuvant ICI. Efficacy and safety of ICI have been examined thoroughly in several studies including patients aged 65 years or older, but real-world data focusing on patients ≥ 75 years is missing [3, 7–9]. In elderly patients, additional comorbidities and aging of the immune system with consecutive immunosenescence might influence the effectiveness of ICI [10, 11]. This might influence not only the tumor response, but also the occurrence of irAE [12]. Aim of this study was to investigate tolerability of adjuvant ICI, i.e. occurrence of irAE and disease-free survival (DFS) in elderly patients with melanoma receiving adjuvant ICI in a real-world-setting.

## Methods

We retrospectively analyzed clinical data of patients with melanoma and adjuvant ICI between January 1st, 2018 and December 20th, 2022 treated at the university dermatological hospital in Tübingen, Germany. Patients were included, if they had received at least one dose of adjuvant ICI with pembrolizumab or nivolumab. This study aimed to examine the outcome and occurrence of irAE of adjuvant ICI in older patients aged ≥ 75 years to younger patients aged 18–74 years. The 75th percentile of age was used to separate the cohort in two cohorts according to age (≥ 75 years and < 75 years).

IrAE were classified according to the Common Terminology Criteria for Adverse Events (CTCAE) classification (version 5), as documented in the patients’ medical records [13]. If no classification was possible according to the records, irAE were classified according to the clinical procedures. Hospitalization with intravenous corticosteroid therapy was classified at least as grade 3 and in the case of life-threatening irAE as grade 4. Endocrinological and pancreatic irAE were classified according to symptoms and clinical management into mild (asymptomatic, ICI continued), moderate (temporary pause of ICI) and severe (discontinuation of ICI, hospitalization and intravenous corticosteroids).

Collection of data and statistical analysis was performed using IBM SPSS Statistics Version 28.0.0.0 (190). *P*-values < 0.05 were considered statistically significant.

Descriptive statistics were used to describe the two cohorts. Differences between the two cohorts of older and younger patients were tested using the Exact Fisher test and the Exact Version of the Chi-Square test for categorical data. Survival analysis was performed by using the log rank test (disease-free survival). Survival curves were calculated by the Kaplan–Meier method. Disease-free survival time was defined as the time between the first cycle of adjuvant ICI and occurrence of progression or death due to melanoma or censored at the last date of follow-up.

This study was approved by the ethics board of the University of Tübingen (648/2022/BO2).

## Results

### Patients’ characteristics

456 patients with adjuvant ICI could be included in the study. Median age was 63 years, interquartile range 53–75 years. 339 patients were aged between 18 and 74 years, 117 patients were ≥ 75 years. Table 1 lists demographic details of the two cohorts.

**Table 1:** Demographic data, type of ICI and outcome

			Patients ≥ 75 years	Patients 18–74 years	*P* value
			*n* (%)	*n* (%)	
			*n* = 117 (100.0)	*n* = 339 (100.0)	
*Gender*					0.660
Female			48 (41.0)	147 (43.4)	
Male			69 (59.0)	192 (56.6)	
Melanoma type					0.382
Cutaneous			88 (75.1)	274 (80.8)	
Acral			10 (8.6)	22 (6.5)	
Uveal			3 (2.6)	4 (1.2)	
Mucosal			7 (6.0)	10 (2.9)	
Occult			9 (7.7)	29 (8.6)	
AJCC (2017) stage at start of ICI**					< 0.001
II B/C			2 (1.7)	7 (2.1)	
III			8 (6.8)	21 (6.2)	
III A			2 (1.7)	54 (15.9)	
III B			31 (26.5)	101 (29.8)	
III C			54 (46.2)	121 (35.7)	
III D			6 (5.1)	4 (1.2)	
IV			14 (12.0)	31 (9.1)	
*Type of adjuvant ICI and schedule at initiation*					0.384
Nivolumab 480 mg q28			98 (83.8)	279 (82.3)	
Nivolumab 240 mg q14			1 (0.9)	8 (2.4)	
Pembrolizumab 400 mg q42			15 (12.8)	49 (14.5)	
Pembrolizumab 200 mg q21			3 (2.6)	3 (0.9)	
Recurrence and follow-up					
Median follow-up (months; IQR)*			21.0 (13.0–37.0)	26.0 (14.0–42.0)	0.016 Median test
Median time to relapse (months, IQR)			9.0 (4.0–18.0)	14.0 (5.0–28.0)	0.007 Median test
Without recurrence at cut-off date**			52 (44.4)	213 (62.8)	< 0.001
Recurrence at cut-off date**			65 (55.6)	126 (37.2)	
Loco-regional			30 (25.6)	64 (18.9)	0.543
Distant			35 (29.9)	62 (18.3)	
Recurrence during adjuvant ICI immunotherapy			37 (31.6)	76 (22.4)	0.651
Recurrence after adjuvant ICI			28 (23.9)	50 (14.7)	
Median duration of adjuvant ICI (months)*			7.0 (3.0–10.0)	10.0 (4.0–11.0)	0.004 Median test
Premature discontinuation of adjuvant ICI**			68 (57.3)	111 (32.1)	< 0.001
Reasons for premature discontinuation**					< 0.001
Progression			31 (27.0)	60 (17.8)	
Adverse events			17 (14.8)	36 (10.7)	
Patient’s preference			7 (6.1)	6 (1.8)	
Other			9 (7.8)	6 (1.8)	

### Application/duration of ICI and recurrence

Table 1 also displays details concerning ICI type, schedule duration and discontinuation rate. Reasons for premature (< 12 months) discontinuation of ICI in the elderly group—listed as “other” in the table—were: general deterioration or hospitalization due to worsening of Eastern Cooperative Oncology Group (ECOG) performance status (*n* = 4), or comorbidities (*n* = 5).

### Safety and adverse events

Table 2 summarizes irAE that occurred during ICI. IrAE of any grade were reported in 73 of the elderly patients (62.4%) and 209 of the younger patients (61.7%). CTCAE grade ≥ 3 were observed in 7 cases (6.0%) of the elderly cohort and 33 cases (10%) of the younger cohort. One patient in the younger cohort died due to ICI-induced myocarditis. No other irAE CTCAE grade 5 occurred. There were 44 patients (37.6%) in the older group and 130 (38.3%) in the younger cohort without any irAE during adjuvant ICI.

**Table 2:** Adverse events during adjuvant immune checkpoint inhibition in *n* = 339 patients 18–74 years and *n* = 117 patients ≥ 75 years [in *n* (%)]

Adverse event	Grade 1–2 / mild to moderate		Grade 3–5 / severe		*P* value
	18–74 years	≥ 75 years	18–74 years	≥ 75 years	
Skin toxicity**	59 (17.4)	35 (30.0)	0	0	< 0.001
Fatigue*	53 (15.7)	8 (6.8)	6 (1.8)	0	0.039
Pancreatitis/elevated lipase/amylase	49 (14.4)	15 (12.9)	3 (0.9)	0	0.655
Myalgia	42 (12.4)	19 (16.3)	2 (0.6)	0	0.622
endocrinological	41 (12.1)	6 (5.1)	3 (0.9)	2 (1.7)	0.790
Hepatitis/ elevated AST/ALT	41 (12.1)	10 (8.5)	4 (1.2)	3 (2.6)	0.548
arthralgia	27 (8.0)	4 (3.4)	1 (0.3)	1 (0.9)	0.255
Colitis/diarrhea*	12 (3.6)	9 (7.7)	7 (2.1)	1 (0.9)	0.026
Pneumonitis	7 (2.1)	4 (3.4)	2 (0.6)	0	0.714
Nephritis/elevated creatine kinase*	7 (2.1)	8 (6.8)	2 (0.6)	0	0.030
Neurological (polyneuropathia/ encephalitis/meningitis)	3 (0.9)	4 (3.4)	1 (0.3)	0	0.251
myocarditis	2 (0.6)	0	2 (0.6)	0	0.845

There was a significantly higher occurrence of skin and nephrological toxicity and diarrhea / colitis in the older cohort. However, the younger cohort experienced fatigue significantly more frequently. In our cohort, skin toxicity was not associated with improved DFS.

### Disease-free survival

**Fig. 1:**
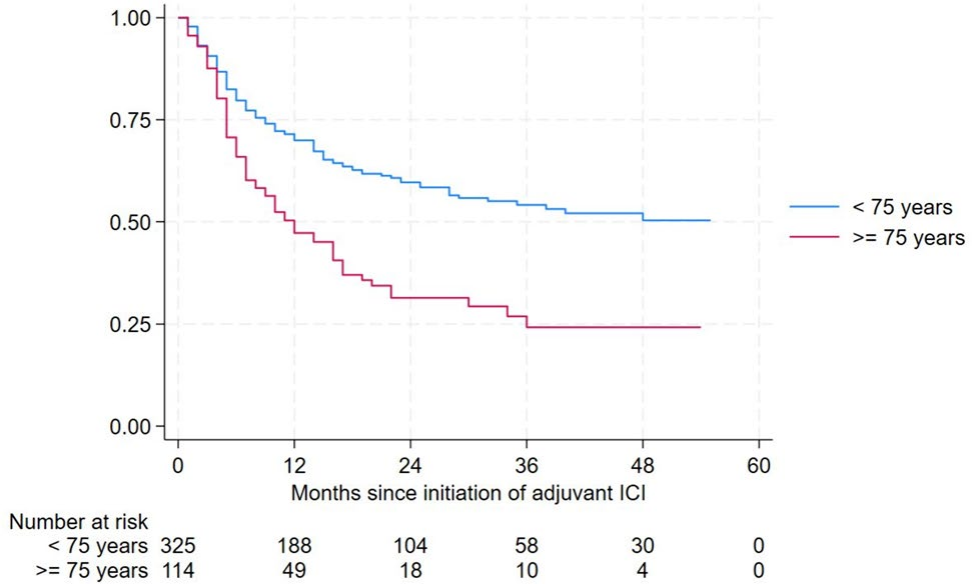
Disease-free survival in melanoma patients receiving adjuvant ICI (*p* < 0.001)

Figure 1 displays DFS in both cohorts with worse DFS in patients > 75 years (*p* < 0.001). The median time between start of ICI and occurrence of metastases was shorter in the elderly group with a median of 9.0 months (interquartile range (IQR) 4.0–18.0 months) and 14.0 months (IQR 5.0–28.0 months) in the younger group (*p* = 0.007). When evaluating separately only stage IIIB and stage IIIC, there was also a markedly reduced DFS for patients aged 75 years or more (supplement 1 and 2). Likewise, we found reduced overall survival (OS) and melanoma specific survival (MSS) in patients older than 75 years (supplement 3 and 4).

## Discussion

Two aspects are important for the implementation of adjuvant ICI in elderly patients ≥ 75 years. The first is safety of adjuvant ICI in elderly patients in terms of irAE, co-morbidities and frailty. The second is the efficacy in elderly patients considering the aging immune system potentially leading to reduced efficacy of adjuvant ICI [11, 14].

Considering the first point, available data is rather scarce with regard to elderly melanoma patients. A subgroup analysis of the KEYNOTE studies in patients with non-small cell lung cancer ≥ 75 years reported 68.5% treatment-related events and 24.2% AEs ≥ grade 3 [15]. Endo et al. [16] found 51.2% adverse events in elderly lung cancer patients treated with PD-1 inhibitors, 17.1% were grade 3 or higher. In our study, we found comparable results. Adjuvant ICI with pembrolizumab or nivolumab was generally well tolerated in the group of elderly patients and 37.1% experienced no irAE at all. The occurrence of grade 3 or 4 irAE was rare with only 7 out of 117 patients (6%) and thus lower than in the group of younger patients (10%). Moreover, no deaths occurred in the elderly due to irAE with adjuvant ICI.

The association between occurrence of irAE and prognosis in melanoma patients has been a subject of debate. On the one hand, several studies found no correlation between irAE and DFS or overall survival (OS) [2, 17, 18]. On the other hand, an association between skin toxicity and improved DFS and OS in advanced melanoma has been observed in a number of other studies [19, 20]. Freeman-Keller et al.[21] also reported a statistically significant association between OS and the occurrence of cutaneous irAE as well as the total number of observed irAE, respectively. Likewise, Eggermont et al. [22] found a longer recurrence-free survival in patients that experienced any treatment-related AE during palliative therapy with pembrolizumab. In our study, elderly patients experienced cutaneous irAE significantly more often. However, there was no correlation between skin toxicity and DFS.

Several studies have shown that elderly melanoma patients in general have a poorer prognosis, as they are more likely to have a higher tumor stage at first diagnosis with higher tumor thickness, a higher mitotic rate and an increased likelihood for ulcerated tumors [23, 24]. While some studies found no age-dependent difference in response rate to anti-PD1 therapy, others reported a shorter DFS in older patients [25, 26]. In a large meta-analysis including more than 35,000 patients, Wu et al. [27] found no significant benefit of ICI in terms of DFS and OS for patients aged 75 years or older. Similar results were obtained in our study. Elderly patients had a shorter disease-free survival and more often relapsed despite ICI than younger patients. Possible explanations include age-related alterations of the immune system, but also premature termination of adjuvant ICI due to deterioration of general condition or personal preference. However, the number of patients with premature termination of adjuvant ICI due to individual preference was low (n < 10). Another limitation is the retrospective character of this study. Only irAE, that had been written in the medical records, have been included. There might have been other irAE in patients that had not been addressed during the consultation with their physician. Nevertheless, there is a high percentage of irAE in this evaluation which correspond to the rates in the literature.

In summary, adjuvant ICI therapy was well tolerated, also in elderly patients and irAE were manageable. In terms of efficacy, it has to be noted that DFS was significantly shorter in the elderly cohort. There was also significantly more skin and nephrological toxicity and more cases of colitis/diarrhea. According to our results, adjuvant ICI should be used with precaution in patients aged ≥ 75 years. Further studies are needed, i.e. concerning adjuvant targeted therapy in terms of efficacy and toxicity.

## Supplementary Information

Below is the link to the electronic supplementary material.

Supplementary file1 (DOCX 10571 kb)
