# Supplementary material for: Efficacy of adjuvant immune checkpoint inhibitors pembrolizumab or nivolumab in melanoma patients ≥ 75 years: results of a real-world cohort including 456 patients
Source: Cancer Immunol Immunother. 2024 Jul 5;73(9):185. doi: 10.1007/s00262-024-03750-1 (PMC11226568; doi:10.1007/s00262-024-03750-1)

Supplement 1 DFS Stage IIIB patients according to age (p=0.003)


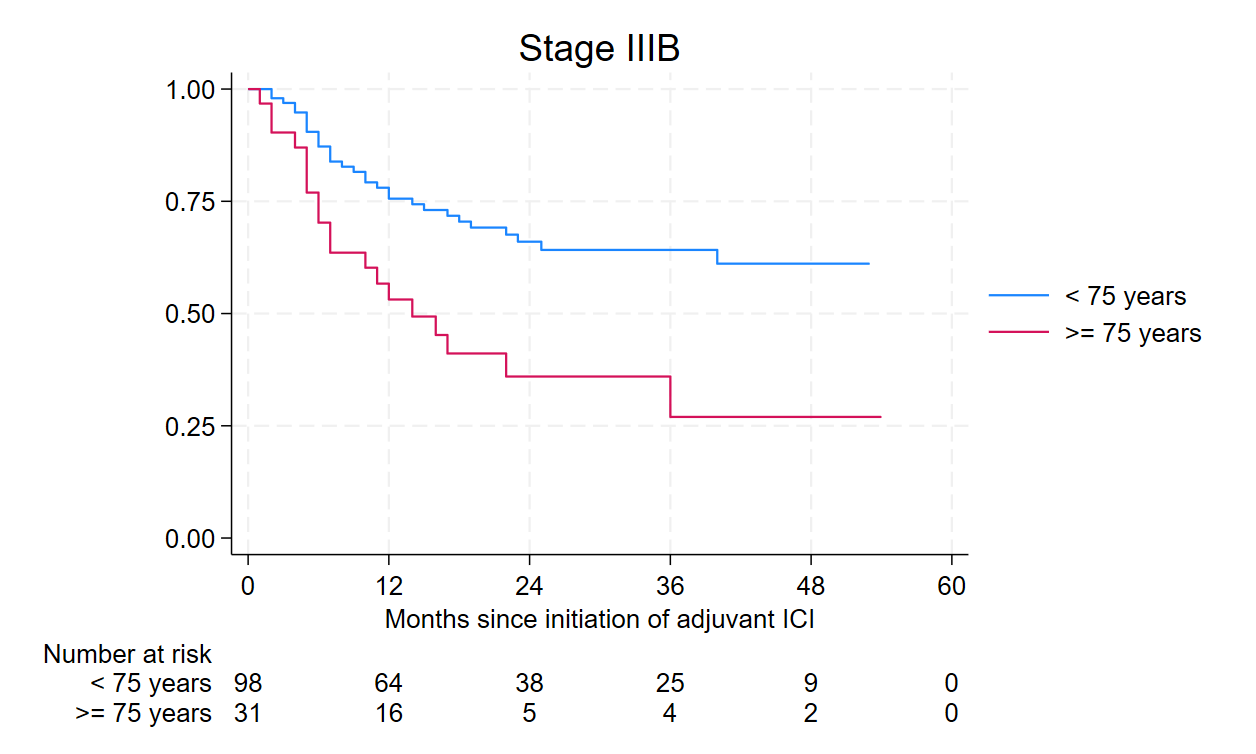


Supplement 2 DFS Stage IIIC patients according to age (p=0.109)


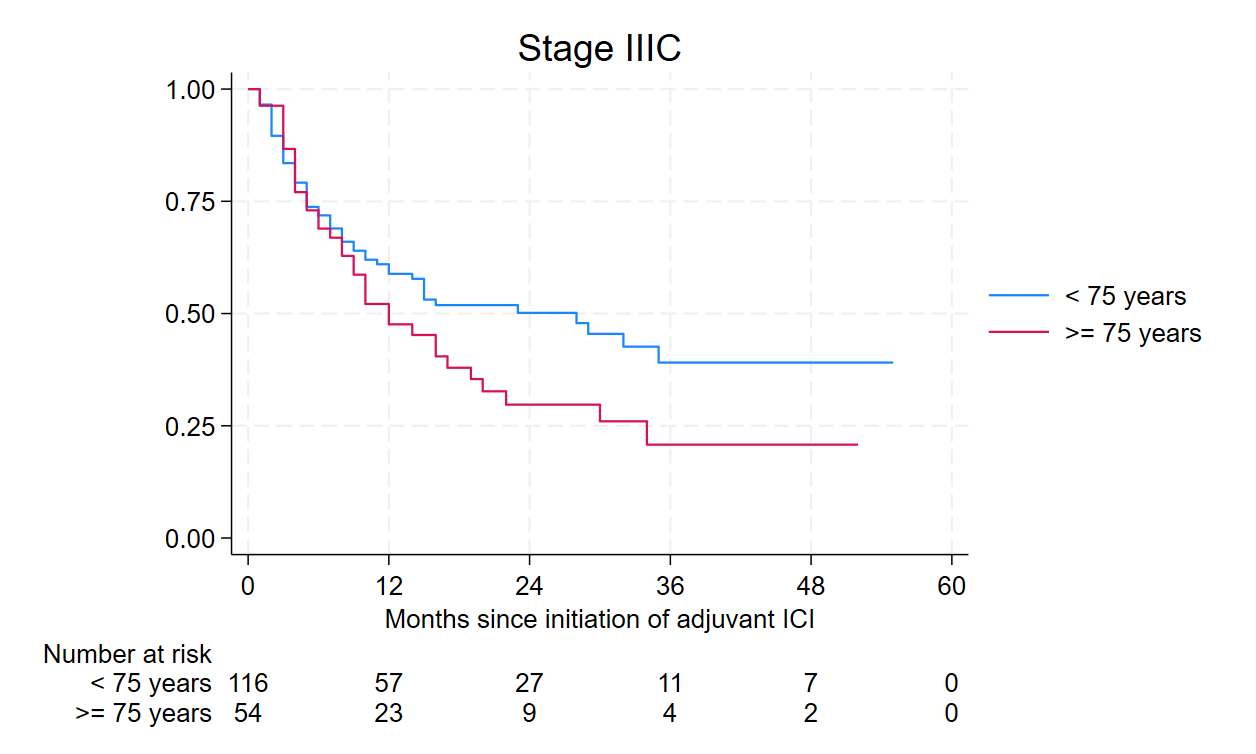


Supplement 3 OS total cohort (p<0.001)


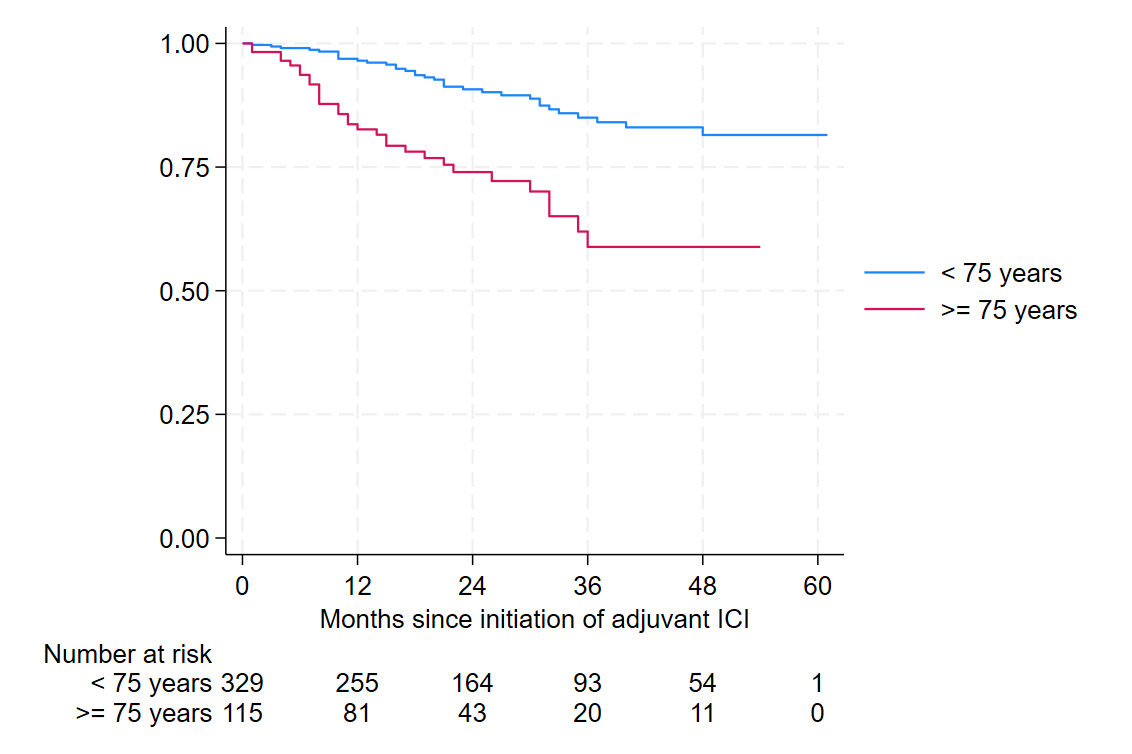


Supplement 4 MSS total cohort (p=0.01)


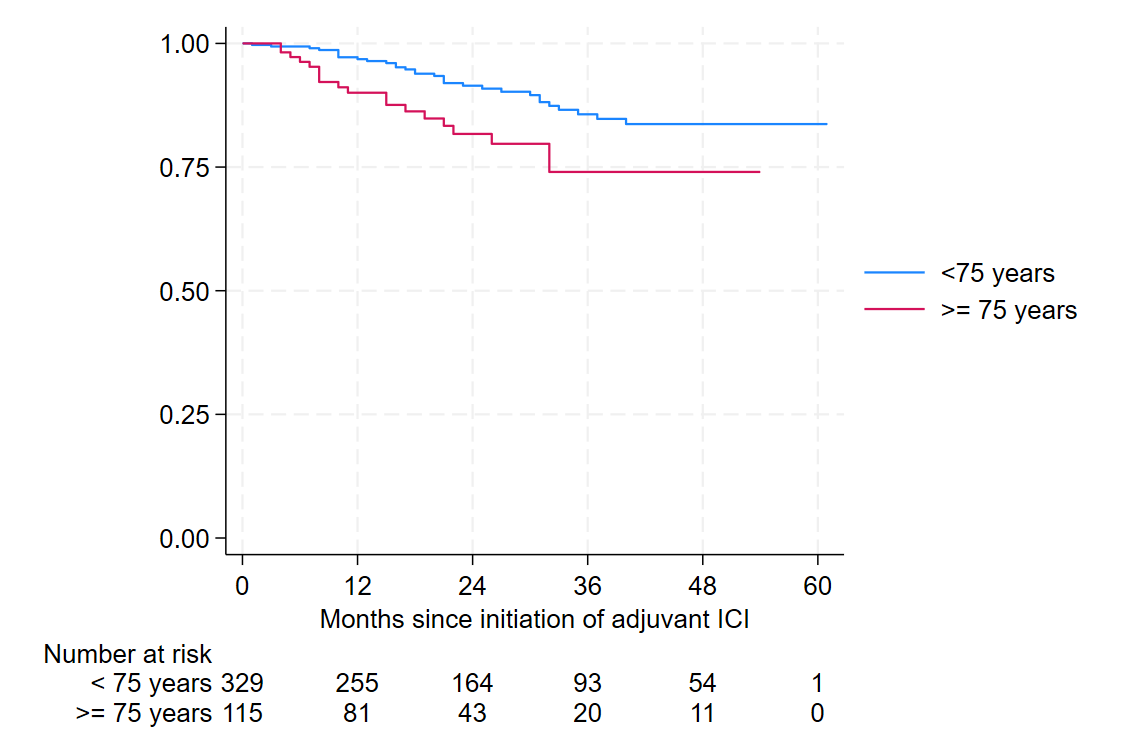

Supplement: Supplementary file 1 — Supplementary file1 (DOCX 10571 kb) [file 262_2024_3750_MOESM1_ESM.docx]
